# RETRACTION: Association Between Diabetic Retinopathy and Diabetic Foot Ulcer in Patients With Diabetes: A Meta‐Analysis

**DOI:** 10.1111/iwj.70312

**Published:** 2025-03-06

## Abstract

**RETRACTION:** LiZ. , WeiJ. , and LuS. , “Association Between Diabetic Retinopathy and Diabetic Foot Ulcer in Patients With Diabetes: A Meta‐Analysis,” International Wound Journal 20, no. 10 (2023): 4077–4082, 10.1111/iwj.14299.37554103 PMC10681479

The above article, published online on 09 August 2023, in Wiley Online Library (http://onlinelibrary.wiley.com/), has been retracted by agreement between the journal Editor in Chief, Professor Keith Harding; and John Wiley & Sons Ltd. Following an investigation by the publisher, all parties have concluded that this article was accepted solely on the basis of a compromised peer review process. The editors have therefore decided to retract the article. The authors did not respond to our notice regarding the retraction.

**RETRACTION:** Z. Li , J. Wei , and S. Lu , “Association Between Diabetic Retinopathy and Diabetic Foot Ulcer in Patients With Diabetes: A Meta‐Analysis,” International Wound Journal 20, no. 10 (2023): 4077–4082, 10.1111/iwj.14299.37554103 PMC10681479

The above article, published online on 09 August 2023, in Wiley Online Library (http://onlinelibrary.wiley.com/), has been retracted by agreement between the journal Editor in Chief, Professor Keith Harding; and John Wiley & Sons Ltd. Following an investigation by the publisher, all parties have concluded that this article was accepted solely on the basis of a compromised peer review process. The editors have therefore decided to retract the article. The authors did not respond to our notice regarding the retraction.

